# Accreditation and resulting clerical duties represent commercial excesses that are ethically and scientifically unacceptable

**Published:** 2018-06

**Authors:** Y Jacquemyn

**Affiliations:** Antwerp University Hospital UZA and Antwerp University UA, ASTARC and Global Health.

**Keywords:** Certification, health care, hospital accreditation, quality, shared decision

## Abstract

It is scientifically and ethically unjustified to continue hospital accreditation organized by commercial organisations such as Joint Commission International (JCI) as these harm patients and health care workers, result in needless excess costs without improved outcome and endanger the future of healthcare. All energy should go to bottom up shared decision making.

At the end of the previous century Evidence Based Medicine (EBM) was introduced as the Holy Grail for improving clinical care, soon hijacked by both governments (to decrease health care expenses) and Big Pharma (to increase financial gains by providing “evidence”) (Greenhalgh et al., 2014 ). Our current century witnessed a major financial crisis followed by the birth of Value Based Medicine (VBM), aiming to increase the value that is derived from the resources available for a population. In this same period clinicians are more and more overwhelmed by hospital administrators and managers who impose top down accreditation by commercially based organizations such as JCI (Joint Commission International), officially to improve patient safety and quality of care. This seems quite an honorable undertaking, but is it really ethically justifiable? The burden caused by administrators and managers by imposing protocols and checklists have been demonstrated to jeopardize the doctor patient relationship, increase costs without better quality and diminish attractiveness of medicine for the future generation (Girbes et al., 2016). In all this, the original foundations of EBM as proposed by David Sacket was completely lost and it was forgotten that EBM “ requires a bottom up approach that integrates the best external evidence with individual clinical expertise and the patients’ choice, it cannot result in slavish, cookbook approaches to individual patient care” (Sackett et al., 1996).

In medical ethics most often a non-normative approach is used based on non-maleficence, respect for autonomy, beneficence and empowerment. The extra amount of time lost in non-EBM supported procedures to comply with commercially based international accreditation (CBIA) is definitely lost for patient-caretaker interaction, resulting in only 13 % of working time reserved for patients, the rest spend to activities without immediate benefit for patients as reported in a recent study from the Netherlands (Schuurman et al., 2018). Furthermore these administrative obligations, filling in endless checklists and other clerical burdens, constitute a major reason for physician burnout, so, clearly CBIA can harm (Schuurman et al., 2018, West et al., 2018). But, then if there is maleficence to medical practitioners, perhaps there is overwhelming beneficence to patients? CBIA has never proven to result in significantly better outcome measures, especially in obstetrics and gynaecology. It does not improve organization and management, specifically when compared to professional accreditation by peers, neither do patient reported outcome measures (the famous PROMS that are now being largely introduced as a newer hype by hospital administrators) relate to CBIA .

A systematic review did not find evidence to support accreditation and certification of hospitals being linked to measurable changes in quality of care (Brubakk et al., 2015). Furthermore it is self evident that the JCI-like accreditation programs necessitate substantial financial and labor investments, which distract finances and healthcare teams from the primary clinical goal of patient care (van Bogaert et al,. 2018). It seems that the only stakeholder with proven benefits is the board of the accreditation company, to consolidate financial profit. These institutions will try to preserve the problem to which they present themselves as the solution.

Is autonomy respected by implementing CBIA rules to physicians, nurses and patients? The introduction of general obligatory check lists and command, check and control systems by their very nature block autonomy and shared decision making. This top down implementation limits empowerment described as worker’s access to relevant information, support and resources but also to learn and grow. It is becoming increasingly clear that patients who do not want to be treated by a fixed and impersonal protocol do encounter difficulties to find a courageous doctor that will support their very personal preferences and choices within the walls of a hospital. Furthermore, as CBIA forces caregivers and receivers alike, to work within narrow limits of allowed practices, there is no room left for empowerment because they create major problems for the accreditation of the institution and are no longer welcome. A few examples: women willing to give birth by an alternative way, be it cesarean on demand: “but please go elsewhere because the cesarean section rate is the most important quality marker for CBIA” or declining fetal monitoring during labour: “please not here we do need something measured every few minutes”.

Anyhow pregnant women demonstrate a (healthy) lack of interest in available quality metrics, which is caused by differences in how women and clinicians/ researchers conceptualize quality. Women are interested in the individual quality of, and the relationship with their personal, obstetrician and they do not believe (wisely) that a hospital’s quality score influences the care they receive (Gourevitch et al., 2017). Luckily, our wise patients know that the presence of protocols, especially in obstetrics, does not lead to detectable improvements in outcomes (Bailit et al., 2015).

Then it can be asked whether the act of complying to CBIA is in accordance with the aim of doing more good than harm, as compared with not complying. For as far as we stand now after years of obsessive obligations we do know that accreditation and certification are positively associated with clinical leadership, the existence of systems for patient safety (not less accidents!) and clinical review, but not with patient outcomes (Sack et al., 2011; Shaw et al., 2014).

Real EBM was a marvelously good idea but this includes making ethical care of the patient a top priority, with individualised evidence characterised by expert judgment rather than mechanical rule following. Such a humane care is only possible by sharing decisions with patients through meaningful conversations, builded on a powerful clinician- patient relationship including autonomy and empowerment of all parties involved (Greenhalgh et al., 2014). To reach this ticking of security lists should be stopped immediately and continuing JCI and other commercial accreditations is no part of this.

The introduction of CBIA is accompanied by the entrance of Dupont’s Dirty dozen: lack of direct communication because one is busy ticking a list, complacency as we are satisfied once all the administration is done even if the patient is bleeding to death, lack of knowledge as in a brainless way guidelines are followed as best practice, distraction caused by endless registration instead of real listening to the patient, lack of teamwork as there is no team left for team interaction, fatigue due to ineffective long working times with minimal patient interaction, lack of resources as money is spent on accreditation companies, pressure to comply with the rules, lack of assertiveness as especially junior doctors do not know any better and do not dare to communicate their own ideas, wants and needs, stress and burnout, lack of awareness on what is really happening with the human being we are supposed to take care of in a mutual interaction, and finally the failure of norms where assumptions are made that the action or procedure is correct without critical thinking. Introduction of the Dirty Dozen leads to less safety and effectiveness.

As health care workers we state that at least 50% of our time is spend on direct patient care, that no new command and control system is introduced before it has been validated to result in an evidence based outcome improvement and finally that patients fully share decisions concerning their health, not hindered nor forced by any commercial accreditation process.

## Declaration conflict of interest:

I declare having major conflicts of interest as a human being wanting to live a creative professional and a healthy personal live.
